# Disparities in HIV clinic care across Europe: findings from the EuroSIDA clinic survey

**DOI:** 10.1186/s12879-016-1685-x

**Published:** 2016-07-20

**Authors:** Jeffrey V. Lazarus, Kamilla Grønborg Laut, Kelly Safreed-Harmon, Lars Peters, Margaret Johnson, Gerd Fätkenheuer, Irina Khromova, Linos Vandekerckhove, Katarzyna Maciejewska, Roxana Radoi, Anna Lisa Ridolfo, Amanda Mocroft

**Affiliations:** CHIP – Centre for Health and Infectious Disease Research, Rigshospitalet, University of Copenhagen, Øster Alle 56, 5. sal, DK-2100, Copenhagen, Denmark; Royal Free and University College Medical School, London, United Kingdom; University Hospital Cologne, Cologne, Germany; Centre for HIV/AIDS and infectious diseases, Kaliningrad, Russian Federation; HIV Translational Research Unit (HTRU), Department of Internal Medicine, Ghent University and Ghent University Hospital, Ghent, Belgium; Department of Infectious, Tropical Diseases and Aquired Immunodeficiencies of Pomeranian Medical University, Szczecin, Poland; Dr. Victor Babes Hospital, Bucharest, Romania; Clinica delle Malattie Infettive, Milan, Italy; Department of Infection and Population Health, University College London, London, United Kingdom

**Keywords:** AIDS, Health care delivery, Health systems, HIV, Europe

## Abstract

**Background:**

Although advances in HIV medicine have yielded increasingly better treatment outcomes in recent years, HIV-positive people with access to antiretroviral therapy (ART) still face complex health challenges. The EuroSIDA Study Group surveyed its clinics to explore regional differences in clinic services.

**Methods:**

The EuroSIDA study is a prospective observational cohort study that began enrolling patients in 1994. In early 2014, we conducted a 59-item survey of the 98 then-active EuroSIDA clinics. The survey covered HIV clinical care and other aspects of patient care. The EuroSIDA East Europe study region (Belarus, Estonia, Lithuania, the Russian Federation and Ukraine) was compared to a “non-East Europe” study region comprised of all other EuroSIDA countries.

**Results:**

A larger proportion of clinics in the East Europe group reported deferring ART in asymptomatic patients until the CD4 cell count dropped below 350 cells/mm^3^ (75 % versus 25 %, *p* = 0.0032). Considerably smaller proportions of East Europe clinics reported that resistance testing was provided before ART initiation (17 % versus 86 %, *p* < 0.0001) and that it was provided upon treatment failure (58 % versus 90 %, *p* = 0.0040). Only 33 % of East Europe clinics reported providing hepatitis B vaccination, compared to 88 % of other clinics (*p* < 0.0001). Only 50 % of East Europe clinics reported having access to direct-acting antivirals for hepatitis C treatment, compared to 89 % of other clinics (*p* = 0.0036). There was significantly less tuberculosis/HIV treatment integration in the East Europe group (27 % versus 84 % *p* < 0.0001) as well as significantly less screening for cardiovascular disease (58 % versus 90 %, *p* = 0.014); tobacco use (50 % versus 93 %, *p* < 0.0001); alcohol consumption (50 % versus 93 %, *p* < 0.0001); and drug use (58 % versus 87 %, *p* = 0.029).

**Conclusions:**

Study findings demonstrate how specific features of HIV clinics differ across Europe. Significantly more East Europe clinics deferred ART in asymptomatic patients for longer, and significantly fewer East Europe clinics provided resistance testing before initiating ART or upon ART failure. The East Europe group of clinics also differed in regard to hepatitis B vaccination, direct-acting antiviral access, tuberculosis/HIV treatment integration and screening for other health issues. There is a need for further research to guide setting-specific decision-making regarding the optimal array of services at HIV clinics in Europe and worldwide.

**Electronic supplementary material:**

The online version of this article (doi:10.1186/s12879-016-1685-x) contains supplementary material, which is available to authorized users.

## Background

Advances in HIV medicine have yielded increasingly better treatment outcomes in recent years, in part because people living with HIV (PHLIV) are now offered more effective and more tolerable antiretroviral therapy (ART) regimens with simpler dosing schedules [1]. Life expectancy has increased greatly for ART-treated PLHIV, and may even be approaching life expectancy in the general population [2, 3]. Nonetheless, HIV remains a major health threat; there were 136,235 new HIV infections reported in the World Health Organization (WHO) European Region in 2013 [4], and HIV was estimated to be the sixth-leading cause of death worldwide in 2010 [5].

Although deaths from HIV are concentrated in resource-limited countries in sub-Saharan Africa and Southeast Asia [6], the disease also continues to claim lives in regions with high levels of treatment coverage. For example, France, Italy and Spain all were estimated to have more than 1000 HIV-related deaths in 2013 [7]. At the same time, non-HIV-related conditions are emerging as prominent health concerns in settings where ART is widely available. A large body of evidence indicates that HIV-positive people are at above-average risk for cardiovascular disease [8] and various non-AIDS-defining cancers [9]. A prospective cohort study of 5185 Spanish PLHIV found that the most common non-AIDS events were psychiatric, liver, kidney, cardiovascular and cancer-related events [10].

This situation raises the question of how the health needs of PLHIV should be addressed beyond the provision of antiretroviral therapy. The global discourse around the response to HIV has emphasised the importance of addressing treatment access barriers such as drug costs, health worker shortages, and laws and policies that discourage marginalised populations from seeking HIV services. Merely having access to ART, however, does not in itself ensure that a person living with HIV will enjoy optimal health outcomes. Following the initiation of ART, virological failure may result from poor adherence, drug resistance, drug toxicity or other factors [11]. Furthermore, achieving viral suppression does not always result in the restoration of the immune system.

Additionally, even in settings where ART is widely available, a multitude of social and institutional factors may influence people’s willingness and ability to adhere to treatment and remain engaged in clinical care. In Valencia, Spain, for example, people who inject drugs (PWID) identified their ongoing drug use as a barrier to adhering fully to ART and reported that a lack of social support hindered adherence as well [12]. In a cohort of African-American men taking ART, adherence was found to be lower among men who experienced stigmatizing attitudes about HIV from members of their social networks [13]. A study of barriers to accessing care among HIV-positive women in 27 countries found that major barriers for women in European countries and Canada included community HIV stigma, lack of employment opportunities and lack of supportive work environments [14].

In light of the array of concerns about the health of HIV-positive people with access to treatment, the EuroSIDA Study Group is exploring whether there are regional differences in health outcomes among its participating clinics and what some of the underlying causes of such differences might be. EuroSIDA has presented evidence of variability across different regions of Europe in initial virologic response to ART [15] and the likelihood of maintaining viral suppression on ART [16], as well as in AIDS-related and non-AIDS-related mortality [17]. Poorer outcomes for the EuroSIDA East Europe study region could not be explained by differences in demographic or HIV-related factors for which we were able to adjust.

These observations led researchers to consider the possible role of factors at the service delivery level. As a preliminary step in pursuing this line of inquiry, we conducted a survey to see whether regional differences could be identified in EuroSIDA clinics in regard to numerous aspects of service provision. The following study presents findings from the first EuroSIDA clinic survey.

## Methods

The EuroSIDA study is a prospective observational cohort study that began enrolling patients in 1994. Details of the study have been published previously [18]. EuroSIDA follows more than 18,000 HIV-positive patients at 108 clinics in 35 European countries, Israel and Argentina. EuroSIDA clinics collect demographic and clinical data from study participants at six-month intervals under the direction of the study coordinating centre, which is based at CHIP, the Centre for Health and Infectious Disease Research (Rigshospitalet, Copenhagen, Denmark). Data collected include CD4 and viral load levels as well as details about antiretroviral (ART) usage, AIDS-defining illnesses, and selected non-AIDS defining clinical events. All study sites have met national ethical requirements and received approval in the countries in which they are located.

In early 2014, our study team conducted a survey of the 97 then-active EuroSIDA clinics (excluding clinics in Argentina). The principal investigator at each EuroSIDA clinic was invited to voluntarily complete the survey. (Principal investigators are medical doctors who are centrally involved with the treatment and management of HIV patients seen at the clinic.) We did not request informed consent from survey respondents because the survey did not ask for identifiable private information about the respondents or any other individuals. The three main sections of the survey asked respondents to answer a total of 59 questions: 31 about clinic and patient characteristics; 22 about HIV clinical care and care of other infectious diseases; and six about non-HIV clinical care. Questions were primarily closed-ended with multiple-choice answers. (The survey is available at www.chip.dk/eurosida/csurvey and in Additional file 1) Content, construct and face validity of the survey were ensured by piloting it in three countries and consulting experts in the field, including the 15 members of the EuroSIDA steering committee. Data were collected and managed through Research Electronic Data Capture (REDcap; http://project-redcap.org), which was hosted at Rigshospitalet. Participants were emailed multiple times, and were called if there was no response.

To inform our understanding of the generalisability of survey findings to the EuroSIDA network as a whole, characteristics of EuroSIDA patients from clinics participating in the survey were compared with characteristics of EuroSIDA patients from non-participating clinics. We included patients from the network if they had been followed up after 1 January 2012, were aged >16 and had undergone CD4 count and viral load testing within 12 months of baseline. Characteristics of persons were summarised using simple summary statistics. Characteristics of persons at participating and non-participating clinics were compared using Wilcoxon signed rank test for continuous variables and chi-squared tests or Fisher’s exact tests when numbers were small for categorical variables.

Previous research has shown EuroSIDA patient outcomes in the South, North and Central Western Europe study regions to be quite similar [16]. Therefore these regions were combined with all other countries apart from the five EuroSIDA East Europe countries to create a “non-East Europe” study region, which then was compared to East Europe (Belarus, Estonia, Lithuania, the Russian Federation and Ukraine) (Table 1). Responses to the clinic survey from these two study regions were compared using simple summary statistics and Wilcoxon signed rank test for continuous variables, while categorical variables were compared using chi-squared tests or Fisher’s exact tests when numbers were small.

**Table 1:** Countries with EuroSIDA clinics participating in the clinic survey

East Europe study region			
Belarus	Lithuania	Ukraine	
Estonia	Russian Federation		
Non-East Europe study region			
*North Europe*	*South Europe*	*West Central Europe*	*East Central Europe*
Denmark	Greece	Austria	Croatia
Finland	Israel	Belgium	Czech Republic
Iceland	Italy	France	Hungary
Ireland	Portugal	Germany	Poland
Netherlands	Spain	Luxembourg	Romania
Norway		Switzerland	Serbia
Sweden			Slovenia
United Kingdom			

All statistical analyses were performed using SAS (Statistical Analysis Software, Cary, NC, USA) version 9.3. A *p*-value of <0.05 was considered statistically significant and all reported tests were 2-sided.

Findings are organised into the following topics in the results section of this paper. After an overview of responding clinics and clinic patients, regional comparisons of survey responses are presented in relation to HIV management, the management of major co-infections, other components of clinical management, and non-clinical support services.

## Results

### Responding clinics and clinic patients

Among 97 currently active EuroSIDA clinics in 35 countries, 81 clinics in 31 countries completed the survey for a response rate of 83.5 %. Most of the responding clinics in the five East Europe countries were government clinics (92 %), while most of the responding clinics in the other countries were university clinics (62 %). Eighty of 81 responding clinics were in urban settings. The median year of the clinic’s establishment was more recent for clinics in the East than clinics in the non-East (1992 [*N* = 12] versus 1985 [*N* = 66]; *p* < 0.0001). Clinics in the East (*N* = 12) reported seeing a median of 2250 HIV-positive patients while clinics in the non-East (*N* = 61) reported seeing a median of 1234 HIV-positive patients, but this difference was not statistically significant.

Two statistically significant differences were found between patient populations at EuroSIDA clinics that responded to the survey and EuroSIDA clinics that did not. Clinics that included more patients into the EuroSIDA cohort were less likely to be non-responders to the survey (adjusted odds ratio [aOR] 0.61 per 50 additional patients, 95 % confidence interval [CI] 0.34–1.08, *p* = 0.091). Clinics with a higher proportion of persons with a prior AIDS diagnosis were more likely to be non-responders (aOR 1.45/10 % higher; 95 % CI 0.97–2.18, *p* = 0.070).

### HIV management

**Fig. 1:**
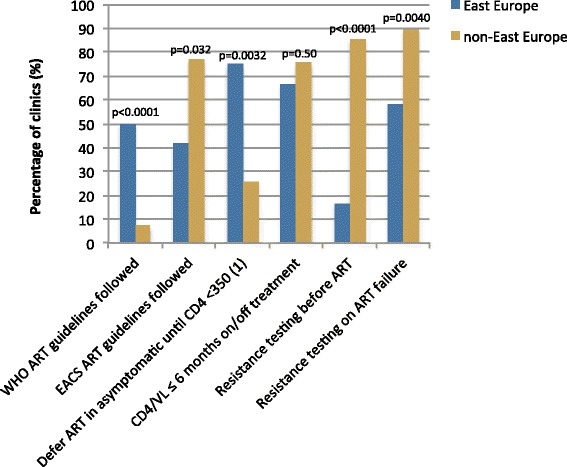
Regional differences in ART treatment and management. (1) data available for *N* = 79, 12 from East Europe and 67 from non-East Europe

A larger proportion of East than non-East clinics reported following the World Health Organization’s HIV treatment guidelines (50 % versus 7 %, *p* < 0.0001) (Fig. 1). At the same time, a smaller proportion of East Europe clinics reported following the European AIDS Clinical Society’s HIV treatment guidelines in comparison to non-East clinics (42 % versus 77 %, *p* = 0.032). A larger proportion of clinics in the East Europe group reported deferring antiretroviral therapy in asymptomatic patients until the CD4 cell count dropped below 350 cells/mm^3^ (75 % versus 25 %, *p* = 0.0032). Two other statistically significant differences between East and non-East clinics related to resistance testing, with considerably smaller proportions of East Europe clinics reporting that resistance testing was provided before the initiation of ART (17 % versus 86 %, *p* < 0.0001) and that it was provided upon treatment failure (58 % versus 90 %, *p* = 0.0040).

### Management of major co-infections

**Fig. 2:**
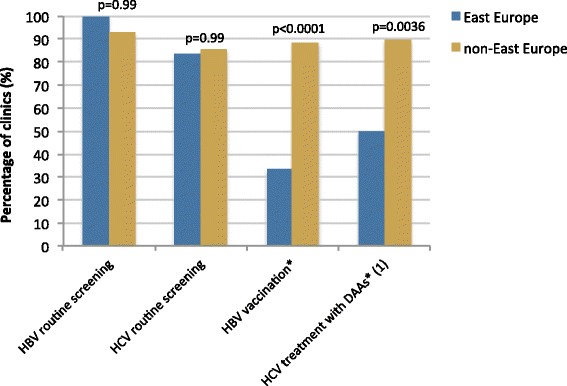
Regional differences in management of hepatitis B and hepatitis C. *including yes and sometimes. (1) data available for *N* = 78, 12 from East Europe and 66 from non-East Europe

The East Europe clinic group and non-East group both reported high levels of routine screening for hepatitis B virus (HBV) and hepatitis C virus (HCV) (Fig. 2). Only 33 % of East Europe clinics reported providing some level of hepatitis B vaccination, compared to 88 % of other clinics (*p* < 0.0001). Only 50 % of East Europe clinics reported having access to direct-acting antivirals (DAAs) for hepatitis C treatment, compared to 89 % of other clinics (*p* = 0.0036).

**Fig. 3:**
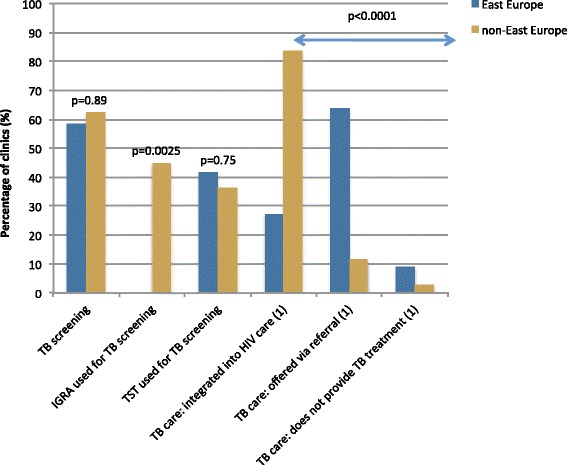
Regional differences in management of tuberculosis. (1) data available for *N* = 78, 11 from East Europe and 67 from non-East Europe

Similar proportions of clinics in the East and non-East groups reported performing tuberculosis (TB) screening (58 % versus 62 %, *p* = 0.89) (Fig. 3). A much smaller proportion of East clinics reported that HIV patients diagnosed with TB received TB treatment integrated into HIV care and treatment (27 % versus 84 %, *p* < 0.0001). Correspondingly, more East Europe clinics reported referring patients with TB to affiliated services for TB treatment (64 % versus 12 %, *p* < 0.0001). One clinic in East Europe reported not providing TB treatment through either of these channels.

### Other components of clinical management

**Fig. 4:**
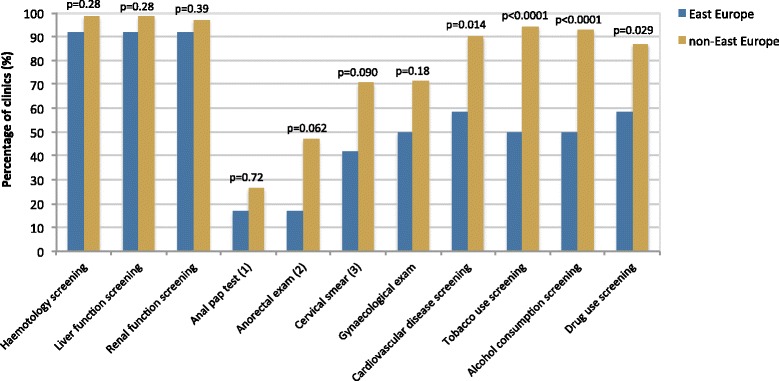
Regional differences in routine screening for other health issues. (1) data available for *N* = 76, 12 from East Europe and 64 from non-East Europe. (2) data available for *N* = 78, 12 from East Europe and 66 from non-East Europe. (3) data available for *N* = 80, 12 from East Europe and 68 from non-East Europe

Clinics in the East and non-East groups reported having similarly high levels of routine screening for haematology, liver function and renal function (Fig. 4). There were lower levels of four other forms of screening: anal pap test, anorectal exam, cervical smear and gynaecological exam. The East Europe group lagged behind the non-East group on all four forms of screening, but these differences were not statistically significant.

There was significantly less screening in East Europe for four health issues: cardiovascular disease (58 % versus 90 %, *p* = 0.014); tobacco use (50 % versus 93 %, *p* < 0.0001); alcohol consumption (50 % versus 93 %, *p* < 0.0001); and drug use (58 % versus 87 %, *p* = 0.029).

### Non-clinical support services

**Fig. 5:**
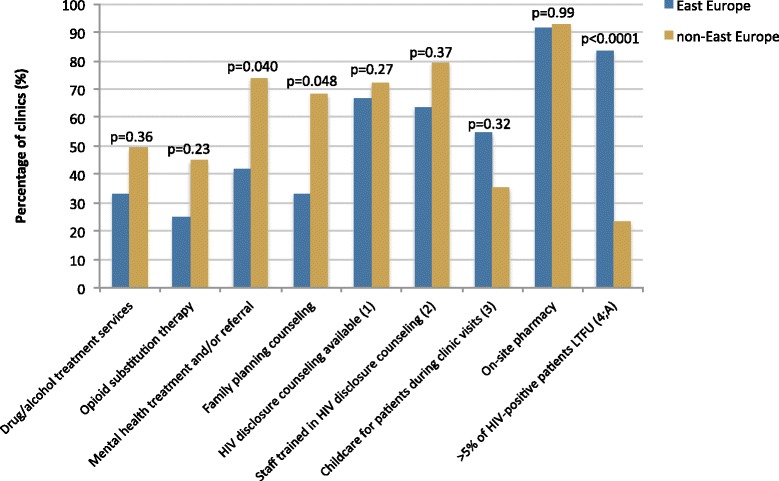
Regional differences in non-clinical support services. (1) data available for *N* = 80, 12 from East Europe and 68 from non-East Europe. (2) data available for *N* = 79, 11 from East Europe and 68 from non-East Europe. (3) data available for *N* = 76, 11 from East Europe and 65 from non-East Europe. (4) data available for *N* = 80, 12 from East Europe and 68 from non-East Europe. (A) Of 27 clinics answering yes, the median % LTFU was 15 (IQR 12–20) in East Europe and 10 (IQR 8–11) in non-East Europe; *p* = 0.021

A diverse array of survey items was used to assess the provision of non-clinical support services (Fig. 5). Drug/alcohol treatment services and opioid substitution therapy were not reported to be available at many clinics either within or outside of East Europe. Although lower proportions of East Europe clinics provided both types of services, the differences were not statistically significant. East Europe clinics also reported non-significantly lower levels of HIV disclosure counselling and staff training for HIV disclosure counselling. High proportions of clinics in both the East and non-East groups were found to have on-site pharmacies while low proportions were found to provide childcare.

Three statistically significant differences were found for non-clinical support services. Eighty-three percent of clinics in East Europe countries reported having loss-to-follow-up levels exceeding 5 % among their HIV-positive patients in the preceding 12 months, compared to 24 % of clinics with greater than 5 % loss-to-follow-up in other countries (*p* < 0.0001). Also, East Europe clinics had lower levels of mental health treatment and/or referral (42 % versus 74 %, *p* = 0.040) as well as lower levels of family planning counselling (33 % versus 68 %, *p* = 0.048).

## Discussion

To our knowledge this is the first large-scale study to compare HIV clinics located in different areas of Europe in terms of a wide range of service delivery features. We chose to compare EuroSIDA study clinics in a group of five countries – Belarus, Estonia, Lithuania, the Russian Federation and Ukraine – to EuroSIDA study clinics elsewhere in Europe because of previous findings of poorer health outcomes for study participants in this region [15–17]. Our findings are consistent with the hypothesis that some clinic characteristics may influence patient outcomes. There were marked differences in how the East Europe group of clinics handled issues such as the initiation of ART in asymptomatic patients and the provision of resistance testing. Furthermore, the East Europe clinics as a whole had a smaller array of services relating to some aspects of viral hepatitis control, tuberculosis control and screening for other health issues.

At the time the clinic survey was conducted, World Health Organization treatment guidelines indicated that ART should always be initiated in HIV-positive people when CD4 cell count levels dropped below 500 cells/mm^3^ [19], while European AIDS Clinical Society guidelines recommended using a lower CD4 threshold of 350 cells/mm^3^ [11]. The clinic survey revealed that at a significantly larger proportion of clinics in East Europe than elsewhere, it was standard practice to delay ART until the CD4 level was below 350 cells/mm^3^. In light of what is now known about early ART initiation having an important protective effect on the immune system, it is reasonable to speculate that having a lower CD4 threshold for initiating ART in asymptomatic patients at East Europe clinics may have contributed to poorer patient outcomes. Patients’ health also may have suffered because of a lack of resistance testing, which was provided by smaller proportions of East Europe clinics both before the initiation of ART and upon treatment failure.

Chronic hepatitis B and hepatitis C disease both have emerged as major health issues for people living with HIV in recent years, with shared routes of transmission for all three diseases accounting for high levels of HIV/HBV coinfection and HIV/HCV coinfection in some populations [20]. In our study, only half of clinics in the East Europe group had access to direct-acting antivirals for hepatitis C treatment, compared to 86 % of clinics elsewhere. DAAs stand apart from earlier generations of HCV treatment for their high cure rates, and the price of the newest, most effective DAAs has raised widespread concern about financial inaccessibility for patients in resource-limited settings and even in resource-rich settings [21, 22]. It is not known whether a lower proportion of EuroSIDA East Europe clinics reported having access to DAAs because of their high cost or for other reasons, but cost seems likely to be a factor as well as patient selection, with many in need being people who inject drugs [23]. The finding points to a need to further investigate differential use of DAAs in HIV/HCV co-infected populations across countries as a step toward determining how liver-related morbidity and mortality in these populations can be reduced.

Study findings for TB screening raise concerns for the entire European region, with fewer than two-thirds of clinics in either study group reporting screening. TB is one of the most common AIDS-indicative diseases diagnosed in the WHO European Region [4], and HIV clinical protocols for the WHO European Region call for all PLHIV to be screened for TB [24]. Fairly large proportions of EuroSIDA clinics in both East Europe and non-East Europe countries appear to not be implementing this guideline. It is possible that the consequences are more pronounced in the East Europe countries, given the high burden of TB and multidrug-resistant TB in those countries [25]. The low level of integration of TB treatment into HIV care and treatment in the East Europe study region – with only 22 % of clinics reporting this to be the case – raises serious concerns in light of what is known about the benefits of an integrated clinical approach to TB/HIV co-infection [26].

The East Europe clinics lagged behind other clinics in regard to screening for cardiovascular disease, tobacco use, alcohol consumption and drug use, all issues with important implications for PLHIV. Cardiovascular disease is a major cause of non-HIV-related mortality in PLHIV populations [27, 28], and people who smoke tobacco have an elevated risk of cardiovascular disease [29]. Alcohol consumption exacerbates the effects on the liver of HBV, HCV and other liver diseases [30], as well as potentially having other negative health effects [31, 32]. While screening for drug use is considered a good practice in many clinical settings, it warrants special consideration in settings where a major pathway for HIV transmission is injecting drug use. This is the case in the East Europe EuroSIDA clinics, with 38 % of the 1370 East Europe study participants who contributed data to this study reporting injecting drug use as their mode of exposure to HIV. In this context, the reported absence of routine screening for drug use at 42 % of East Europe clinics stands out as an issue that may have important implications for patients’ health.

Taken together, our study findings raise important questions regarding whether the availability of a range of services at clinics caring for PLHIV might have an impact on morbidity and mortality. At a time when antiretroviral therapy is widely available in high-income countries and is becoming increasingly available in low- and middle-income countries, these questions are important to take up since they reflect a growing awareness that antiretroviral therapy alone is not sufficient to safeguard the long-term health of PLHIV. Surprisingly, in light of the advanced state of HIV management in some regards, there appears to not be a large evidence base regarding which services a clinic should provide to its HIV-positive patients. The comprehensive HIV care model, with a healthcare team coordinating primary care, HIV care, and other specialist care as well as psychosocial and social services, has long been championed in the United States [33] and has likely influenced the development of many multifaceted HIV clinical initiatives worldwide. However, there is scant evidence regarding the relationship between specific HIV clinic characteristics and patient health outcomes.

A 2006 Cochrane review of studies that assessed various elements of the “setting and organisation of care” for PLHIV found an association between case management and decreased mortality, but concluded that the small evidence base in this field was not sufficient to determine an ideal set of clinic characteristics [34]. Since the publication of the Cochrane review, little new evidence has emerged. A 2009 retrospective cohort study of PLHIV at health facilities for US military veterans found that patients attending clinics that integrated hepatitis, psychiatric, psychological and social services into HIV clinical management were more than three times as likely to achieve viral suppression on ART than patients attending HIV clinics without integrated services [35].

A key challenge in conceptualising and conducting meaningful research in this domain is the setting-specific nature of how health services are organised, funded, managed and governed. For example, the issue of whether or how to integrate HIV clinical services with other clinical services has been highlighted particularly in the context of efforts to provide HIV care in severely resource-constrained settings, with studies in sub-Saharan Africa and elsewhere examining different service delivery models [36] and undertaking different forms of service integration in order to provide HIV services alongside other services such as tuberculosis management [37] and reproductive health care [38]. This research may be of limited value to health system decision-makers in settings with much higher physician-patient ratios or with strong referral systems linking long-established HIV clinics to other services. Clearly there are major differences in regard to which health service delivery models are predominant in different countries, with some differences largely attributable to resource limitations in poorer countries.

Nonetheless, the identification of key elements of successful patient management in diverse settings presents opportunities to explore ways in which these elements may or may not be uniquely dependent on specific features of the local and national health system and the social, political and economic context. It is entirely possible that some service delivery innovations in sub-Saharan Africa may be relevant to health systems in Western Europe, and vice versa. By calling attention to ways in which two regional groupings of clinics for PLHIV in Europe differ from each other, the EuroSIDA clinic survey findings serve as an invitation for clinics and regions with suboptimal patient outcomes to investigate whether the adoption of practices from other settings may be beneficial. Thus the lack of generalisability of a study of the characteristics of health service delivery in a specific group of clinics may be offset by its potential to highlight issues warranting further setting-specific research including operational research on service delivery modifications in “real world” clinic populations.

In sum, our study makes a unique contribution to the issue of HIV management by exploring whether the characteristics of clinics vary across two groups of European countries that have had marked differences in patient outcomes in a large observational study cohort. The identification of some statistically significant differences in clinic characteristics cannot be interpreted as evidence that one or more of those differences is causing the observed differences in patient outcomes. However, findings suggest that it may be beneficial to conduct further research on the potential health impact of clinic characteristics such as the CD4 threshold for ART initiation; ART resistance testing practices; HCV treatment standards; and the provision of screening for non-HIV-related conditions including alcohol and drug dependency. Policy-makers should consider research on service delivery factors alongside other types of research on biomedical factors, health system factors and the influence of the social, political and economic context in order to optimally configure health care for PLHIV at the national and subnational levels.

### Limitations

This study has the following limitations. While our survey addressed major aspects of the clinical and non-clinical care of people living with HIV, there may be other aspects of patient care that have implications for patient outcomes. The study utilises survey data that were reported by clinic representatives whose responses to questions might not reflect what actually happens in clinical practice. Clinical decision-making about some of the issues addressed in our study can be expected to vary in accordance with individual physicians’ preferences as well as patient characteristics such as the nature of symptoms and severity of disease. Furthermore, study findings reflect self-reporting, and it is not possible to verify the accuracy of the information reported. Respondents may have made errors or altered their responses to suggest a higher level of compliance with guidelines. The survey was conducted in English, which may have affected how questions were interpreted by some respondents. Findings have limited generalisability because the EuroSIDA clinics constitute only a small proportion of HIV clinics in European countries. Generalisability is also affected by key characteristics of the responding EuroSIDA clinics: half were university clinics, almost half were government-affiliated, and many were located in capital cities. Practices at these clinics are therefore not necessarily representative of HIV management in the European region overall.

## Conclusions

Our study findings raise important questions about how specific features of HIV clinics might contribute to the geographical differences in patient outcomes in the EuroSIDA study cohort. Significantly more East Europe clinics deferred ART in asymptomatic patients until the CD4 cell count dropped below 350 cells/mm^3^, and significantly fewer East Europe clinics provided resistance testing before initiating ART or upon ART failure. The East Europe group of clinics also compared unfavourably to the non-East Europe group in regard to HBV vaccination, DAA access, TB/HIV treatment integration and screening for cardiovascular disease, smoking, alcohol use and drug use. There is a need for further research to guide setting-specific decision-making regarding the optimal array of services at HIV clinics in Europe and worldwide.

## Abbreviations

ART, antiretroviral therapy; CHIP, The Centre for Health and Infectious Disease Research; DAAs, direct acting antivirals; HBV, hepatitis B virus; HCV, hepatitis C virus; HIV, Human immunodeficiency virus; PHLIV, People living with HIV; TB, Tuberculosis; WHO, World Health Organization

## Additional files

Additional file 1: EuroSIDA Clinic Survey. (DOCX 79 kb) Additional file 2: The multi-centre EuroSIDA study group. (DOCX 29 kb) Additional file 3: Ethics committees by country. (PDF 110 kb)
