# Supplementary material for: Disparities in HIV clinic care across Europe: findings from the EuroSIDA clinic survey
Source: BMC Infect Dis. 2016 Jul 20;16:335. doi: 10.1186/s12879-016-1685-x (PMC4955207; doi:10.1186/s12879-016-1685-x)
Supplement: Additional file 1: — EuroSIDA Clinic Survey. (DOCX 79 kb) [file 12879_2016_1685_MOESM1_ESM.docx]

**[TITLE PAGE]**

**Disparities in HIV clinic care across Europe: findings from the EuroSIDA clinic survey**

**Jeffrey V Lazarus^1^**^§^**, Kamilla Grønborg Laut^1^, Kelly Safreed-Harmon^1^, Lars Peters^1^, Margaret Johnson^2^, Gerd Fätkenheuer^3^, Irina Khromova^4^, Linos Vandekerckhove^5^, Katarzyna Maciejewska^6^, Roxana Radoi^7^, Anna Lisa Ridolfo^8^, Amanda Mocroft^9^**

1. CHIP – Centre for Health and Infectious Disease Research, Rigshospitalet, University of Copenhagen, Denmark

2. Royal Free and University College Medical School, London, United Kingdom

3. University Hospital Cologne, Cologne, Germany

4. Centre for HIV/AIDS and infectious diseases, Kaliningrad, Russian Federation

5. HIV Translational Research Unit (HTRU), Department of Internal Medicine, Ghent University and Ghent University Hospital, Ghent, Belgium

6. Department of Infectious, Tropical Diseases and Aquired Immunodeficiencies of Pomeranian Medical University, Szczecin, Poland

7. Dr. Victor Babes Hospital, Bucharest, Romania

8. Clinica delle Malattie Infettive, Milan, Italy

9. Department of Infection and Population Health, University College London, London, United Kingdom

**Word count:** 4271

**Keywords:** AIDS, Health care delivery, Health systems, HIV, Europe

**Corresponding author:**

Jeffrey V Lazarus

CHIP, Rigshospitalet, University of Copenhagen

Øster Alle 56, 5. sal

DK-2100 Copenhagen Oe

Denmark

Tel: +45 35 45 5757

JVL: Jeffrey.Lazarus@regionh.dk

KGL: kamilla.groenborg.laut.01@regionh.dk

KSH: kelly@safreed-harmon.com

LP: [Lars.Peters@regionh.dk](mailto:Lars.Peters@regionh.dk)

MJ: margaret.johnson1@nhs.net

GF: [g.faetkenheuer@uni-koeln.de](mailto:g.faetkenheuer@uni-koeln.de)

IK: [iekhromova@mail.ru](mailto:iekhromova@mail.ru)

LV: [linos.vandekerckhove@ugent.be](mailto:linos.vandekerckhove@ugent.be)

KM: kafu1@wp.pl

RR: [dr_roxana_radoi@yahoo.com](mailto:dr_roxana_radoi@yahoo.com)

ALR: [annalisa.ridolfo@unimi.it](mailto:annalisa.ridolfo@unimi.it)

AM: [a.mocroft@ucl.ac](mailto:a.mocroft@ucl.ac).uk

**Abstract**

Background

Although advances in HIV medicine have yielded increasingly better treatment outcomes in recent years, HIV-positive people with access to antiretroviral therapy (ART) still face complex health challenges. The EuroSIDA Study Group surveyed its clinics to explore regional differences in clinic services.

Methods

The EuroSIDA study is a prospective observational cohort study that began enrolling patients in 1994. In early 2014, we conducted a 59-item survey of the 98 then-active EuroSIDA clinics. The survey covered HIV clinical care and other aspects of patient care. The EuroSIDA East Europe study region (Belarus, Estonia, Lithuania, the Russian Federation and Ukraine) was compared to a “non-East Europe” study region comprised of all other EuroSIDA countries.

Results

A larger proportion of clinics in the East Europe group reported deferring ART in asymptomatic patients until the CD4 cell count dropped below 350 cells/mm^3^ (75% versus 25%, p=0.0032). Considerably smaller proportions of East Europe clinics reported that resistance testing was provided before ART initiation (17% versus 86%, p<0.0001) and that it was provided upon treatment failure (58% versus 90%, p=0.0040). Only 33% of East Europe clinics reported providing HBV vaccination, compared to 88% of other clinics (p<0.0001). Only 50% of East Europe clinics reported having access to direct-acting antivirals for HCV treatment, compared to 89% of other clinics (p=0.0036). There was significantly less TB/HIV treatment integration in the East Europe group (27% versus 84% p<0.0001) as well as significantly less screening for cardiovascular disease (58% versus 90%, p=0.014); tobacco use (50% versus 93%, p<0.0001); alcohol consumption (50% versus 93%, p<0.0001); and drug use (58% versus 87%, p=0.029).

Conclusions

Study findings demonstrate how specific features of HIV clinics differ across Europe. Significantly more East Europe clinics deferred ART in asymptomatic patients for longer, and significantly fewer East Europe clinics provided resistance testing before initiating ART or upon ART failure. The East Europe group of clinics also differed in regard to HBV vaccination, DAA access, TB/HIV treatment integration and screening for other health issues. There is a need for further research to guide setting-specific decision-making regarding the optimal array of services at HIV clinics in Europe and worldwide.

**BACKGROUND**

Advances in HIV medicine have yielded increasingly better treatment outcomes in recent years, in part because people living with HIV (PHLIV) are now offered more effective and more tolerable antiretroviral therapy (ART) regimens with simpler dosing schedules.[1] Life expectancy has increased greatly for ART-treated PLHIV, and may even be approaching life expectancy in the general population.[2,3] Nonetheless, HIV remains a major health threat; there were 136,235 new HIV infections reported in the World Health Organization (WHO) European Region in 2013,[4] and HIV was estimated to be the sixth-leading cause of death worldwide in 2010.[5]

Although deaths from HIV are concentrated in resource-limited countries in sub-Saharan Africa and Southeast Asia,[6] the disease also continues to claim lives in regions with high levels of treatment coverage. For example, France, Italy and Spain all were estimated to have more than 1,000 HIV-related deaths in 2013.[7] At the same time, non-HIV-related conditions are emerging as prominent health concerns in settings where ART is widely available. A large body of evidence indicates that HIV-positive people are at above-average risk for cardiovascular disease [8] and various non-AIDS-defining cancers.[9] A prospective cohort study of 5185 Spanish PLHIV found that the most common non-AIDS events were psychiatric, liver, kidney, cardiovascular and cancer-related events.[10]

This situation raises the question of how the health needs of PLHIV should be addressed beyond the provision of antiretroviral therapy. The global discourse around the response to HIV has emphasised the importance of addressing treatment access barriers such as drug costs, health worker shortages, and laws and policies that discourage marginalised populations from seeking HIV services. Merely having access to ART, however, does not in itself ensure that a person living with HIV will enjoy optimal health outcomes. Following the initiation of ART, virological failure may result from poor adherence, drug resistance, drug toxicity or other factors.[11] Furthermore, achieving viral suppression does not always result in the restoration of the immune system.

Additionally, even in settings where ART is widely available, a multitude of social and institutional factors may influence people’s willingness and ability to adhere to treatment and remain engaged in clinical care. In Valencia, Spain, for example, people who inject drugs (PWID) identified their ongoing drug use as a barrier to adhering fully to ART and reported that a lack of social support hindered adherence as well.[12] In a cohort of African-American men taking ART, adherence was found to be lower among men who experienced stigmatizing attitudes about HIV from members of their social network.[13] A study of barriers to accessing care among HIV-positive women in 27 countries found that major barriers for women in European countries and Canada included community HIV stigma, lack of employment opportunities and lack of supportive work environments.[14]

In light of the array of concerns about the health of HIV-positive people with access to treatment, the EuroSIDA Study Group is exploring whether there are regional differences in health outcomes among its participating clinics and what some of the underlying causes of such differences might be. EuroSIDA has presented evidence of variability across different regions of Europe in initial virologic response to ART [15] and the likelihood of maintaining viral suppression on ART,[16] as well as in AIDS-related and non-AIDS-related mortality.[17] Poorer outcomes for the EuroSIDA East Europe study region could not be explained by differences in demographic or HIV-related factors for which we were able to adjust.

These observations led researchers to consider the possible role of factors at the service delivery level. As a preliminary step in pursuing this line of inquiry, we conducted a survey to see whether regional differences could be identified in EuroSIDA clinics in regard to numerous aspects of service provision. The following study presents findings from the first EuroSIDA clinic survey.

**METHODS**

The EuroSIDA study is a prospective observational cohort study that began enrolling patients in 1994. Details of the study have been published previously.[18] EuroSIDA follows more than 18,000 HIV-positive patients at 108 clinics in 35 European countries, Israel and Argentina. EuroSIDA clinics collect demographic and clinical data from study participants at six-month intervals under the direction of the study coordinating centre, which is based at CHIP, the Centre for Health and Infectious Disease Research (Rigshospitalet, Copenhagen, Denmark). Data collected include CD4 and viral load levels as well as details about antiretroviral (ART) usage, AIDS-defining illnesses, and selected non-AIDS defining clinical events. All study sites have met national ethical requirements and received approval in the countries in which they are located.

In early 2014, our study team conducted a survey of the 97 then-active EuroSIDA clinics (excluding clinics in Argentina). The principal investigator at each EuroSIDA clinic was invited to voluntarily complete the survey. (Principal investigators are medical doctors who are centrally involved with the treatment and management of HIV patients seen at the clinic.) We did not request informed consent from survey respondents because the survey did not ask for identifiable private information about the respondents or any other individuals. The three main sections of the survey asked respondents to answer a total of 59 questions: 31 about clinic and patient characteristics; 22 about HIV clinical care and care of other infectious diseases; and six about non-HIV clinical care. Questions were primarily closed-ended with multiple-choice answers. (The survey is available at [www.chip.dk/eurosida/csurvey](http://www.chip.dk/eurosida/csurvey) and in Additional File 1.) Content, construct and face validity of the survey were ensured by piloting it in three countries and consulting experts in the field, including the 15 members of the EuroSIDA steering committee. Data were collected and managed through Research Electronic Data Capture (REDcap; <http://project-redcap.org>), which was hosted at Rigshospitalet. Participants were emailed multiple times, and were called if there was no response.

To inform our understanding of the generalisability of survey findings to the EuroSIDA network as a whole, characteristics of EuroSIDA patients from clinics participating in the survey were compared with characteristics of EuroSIDA patients from non-participating clinics. We included patients from the network if they had been followed up after 1 January 2012, were aged >16 and had undergone CD4 count and viral load testing within 12 months of baseline. Characteristics of persons were summarised using simple summary statistics. Characteristics of persons at participating and non-participating clinics were compared using Wilcoxon signed rank test for continuous variables and chi-squared tests or Fisher’s exact tests when numbers were small for categorical variables.

Previous research has shown EuroSIDA clinics in the South, North and Central Western Europe study regions to be quite similar.[19] Therefore these regions were combined with all other countries apart from the five EuroSIDA East Europe countries to create a “non-East Europe” study region, which then was compared to East Europe (Belarus, Estonia, Lithuania, the Russian Federation and Ukraine) (Table 1). Responses to the clinic survey from Eastern and non-Eastern Europe clinics were compared using simple summary statistics and Wilcoxon signed rank test for continuous variables, while categorical variables were compared using chi-squared tests or Fisher’s exact tests when numbers were small.

All statistical analyses were performed using SAS (Statistical Analysis Software, Cary, NC, USA) version 9.3. A p-value of <0.05 was considered statistically significant and all reported tests were 2-sided.

**Table 1. Countries with EuroSIDA clinics participating in the clinic survey**

| **East Europe study region** | | | |
| --- | --- | --- | --- |
| Belarus | Lithuania | Ukraine |  |
| Estonia | Russian Federation |  |  |
|  |  |  |  |
| **Non-East Europe study region** | | | |
| *North Europe* | *South Europe* | *West Central Europe* | *East Central Europe* |
| Denmark | Greece | Austria | Croatia |
| Finland | Israel | Belgium | Czech Republic |
| Iceland | Italy | France | Hungary |
| Ireland | Portugal | Germany | Poland |
| Netherlands | Spain | Luxembourg | Romania |
| Norway |  | Switzerland | Serbia |
| Sweden |  |  | Slovenia |
| United Kingdom |  |  |  |
|  |  |  |  |

Findings are organised into the following topics in the results section of this paper. After an overview of responding clinics and clinic patients, regional comparisons of survey responses are presented in relation to HIV management, the management of major co-infections, other components of clinical management, and non-clinical support services.

**RESULTS**

**Responding clinics and clinic patients**

Among 97 currently active EuroSIDA clinics in 35 countries, 81 clinics in 31 countries completed the survey for a response rate of 83.5%. Most of the responding clinics in the five East Europe countries were government clinics (92%), while most of the responding clinics in the other countries were university clinics (62%). Eighty of 81 responding clinics were in urban settings. The median year of the clinic’s establishment was more recent for clinics in the East than clinics in the non-East (1992 [N=12] versus 1985 [N=66]; p<0.0001). Clinics in the East (N=12) reported seeing a median of 2,250 HIV-positive patients while clinics in the non-East (N=61) reported seeing a median of 1,234 HIV-positive patients, but this difference was not statistically significant.

Two statistically significant differences were found between patient populations at EuroSIDA clinics that responded to the survey and EuroSIDA clinics that did not. Clinics that included more patients into the EuroSIDA cohort were less likely to be non-responders to the survey (adjusted odds ratio [aOR] 0.61 per 50 additional patients, 95% confidence interval [CI] 0.34–1.08, p=0.091). Clinics with a higher proportion of persons with a prior AIDS diagnosis were more likely to be non-responders (aOR 1.45/10% higher; 95% CI 0.97–2.18, p=0.070).

**HIV management**

A larger proportion of East than non-East clinics reported following the World Health Organization’s HIV treatment guidelines (50% versus 7%, p<0.0001) (Figure 1). At the same time, a smaller proportion of East Europe clinics reported following the European AIDS Clinical Society’s HIV treatment guidelines in comparison to non-East clinics (42% versus 77%, p=0.032). A larger proportion of clinics in the East Europe group reported deferring antiretroviral therapy in asymptomatic patients until the CD4 cell count dropped below 350 cells/mm^3^ (75% versus 25%, p=0.0032). Two other statistically significant differences between East and non-East clinics related to resistance testing, with considerably smaller proportions of East Europe clinics reporting that resistance testing was provided before the initiation of ART (17% versus 86%, p<0.0001) and that it was provided upon treatment failure (58% versus 90%, p=0.0040).

**Management of major co-infections**

The East Europe clinic group and non-East group both reported high levels of routine screening for hepatitis B virus (HBV) and hepatitis C virus (HCV) (Figure 2). Only 33% of East Europe clinics reported providing some level of HBV vaccination, compared to 88% of other clinics (p<0.0001). Only 50% of East Europe clinics reported having access to direct-acting antivirals (DAAs) for HCV treatment, compared to 89% of other clinics (p=0.0036).

Similar proportions of clinics in the East and non-East groups reported performing tuberculosis (TB) screening (58% versus 62%, p=0.89) (Figure 3). A much smaller proportion of East clinics reported that HIV patients diagnosed with TB received TB treatment integrated into HIV care and treatment (27% versus 84%, p<0.0001). Correspondingly, more East Europe clinics reported referring patients with TB to affiliated services for TB treatment (64% versus 12%, p<0.0001). One clinic in East Europe reported not providing TB treatment through either of these channels.

**Other components of clinical management**

Clinics in the East and non-East groups reported having similarly high levels of routine screening for haematology, liver function and renal function (Figure 4). There were lower levels of four other forms of screening: anal pap test, anorectal exam, cervical smear and gynaecological exam. The East Europe group lagged behind the non-East group on all four forms of screening, but these differences were not statistically significant.

There was significantly less screening in East Europe for four health issues: cardiovascular disease (58% versus 90%, p=0.014); tobacco use (50% versus 93%, p<0.0001); alcohol consumption (50% versus 93%, p<0.0001); and drug use (58% versus 87%, p=0.029).

**Non-clinical support services**

A diverse array of survey items was used to assess the provision of non-clinical support services (Figure 5). Drug/alcohol treatment services and opioid substitution therapy were not reported to be available at many clinics either within or outside of East Europe. Although lower proportions of East Europe clinics provided both types of services, the differences were not statistically significant. East Europe clinics also reported non-significantly lower levels of HIV disclosure counselling and staff training for HIV disclosure counselling. High proportions of clinics in both the East and non-East groups were found to have on-site pharmacies while low proportions were found to provide childcare.

Three statistically significant differences were found for non-clinical support services. Eighty-three percent of clinics in East Europe countries reported having loss-to-follow-up levels exceeding 5% among their HIV-positive patients in the preceding 12 months, compared to 24% of clinics with greater than 5% loss-to-follow-up in other countries (p<0.0001). Also, East Europe clinics had lower levels of mental health treatment and/or referral (42% versus 74%, p=0.040) as well as lower levels of family planning counselling (33% versus 68%, p=0.048).

**DISCUSSION**

To our knowledge this is the first large-scale study to compare HIV clinics located in different areas of Europe in terms of a wide range of service delivery features. We chose to compare EuroSIDA study clinics in a group of five countries – Belarus, Estonia, Lithuania, the Russian Federation and Ukraine – to EuroSIDA study clinics elsewhere in Europe because of previous findings of poorer health outcomes for study participants in this region.[15–17] Our findings are consistent with the hypothesis that some clinic characteristics may influence patient outcomes. There were marked differences in how the East Europe group of clinics handled issues such as the initiation of ART in asymptomatic patients and the provision of resistance testing. Furthermore, the East Europe clinics as a whole had a smaller array of services relating to some aspects of viral hepatitis control, tuberculosis control and screening for other health issues.

At the time the clinic survey was conducted, World Health Organization treatment guidelines indicated that ART should always be initiated in HIV-positive people when CD4 cell count levels dropped below 500 cells/mm^3^,[20] while European AIDS Clinical Society guidelines recommended using a lower CD4 threshold of 350 cells/mm^3^.[11] The clinic survey revealed that at a significantly larger proportion of clinics in East Europe than elsewhere, it was standard practice to delay ART until the CD4 level was below 350 cells/mm^3^. In light of what is now known about early ART initiation having an important protective effect on the immune system, it is reasonable to speculate that having a lower CD4 threshold for initiating ART in asymptomatic patients at East Europe clinics may have contributed to poorer patient outcomes. Patients’ health also may have suffered because of a lack of resistance testing, which was provided by

smaller proportions of East Europe clinics both before the initiation of ART and upon treatment failure.

Chronic hepatitis B and hepatitis C disease both have emerged as major health issues for people living with HIV in recent years, with shared routes of transmission for all three diseases accounting for high levels of HIV/HBV coinfection and HIV/HCV coinfection in some populations.[21] In our study, only half of clinics in the East Europe group had access to direct-acting antivirals for HCV treatment, compared to 86% of clinics elsewhere. DAAs stand apart from earlier generations of HCV treatment for their high cure rates, and the price of the newest, most effective DAAs has raised widespread concern about financial inaccessibility for patients in resource-limited settings and even in resource-rich settings.[22,23] It is not known whether a lower proportion of EuroSIDA East Europe clinics reported having access to DAAs because of their high cost or for other reasons, but cost seems likely to be a factor as well as patient selection, with many in need being people who inject drugs.[24] The finding points to a need to further investigate differential use of DAAs in HIV/HCV co-infected populations across countries as a step toward determining how liver-related morbidity and mortality in these populations can be reduced.

Study findings for TB screening raise concerns for the entire European region, with fewer than two-thirds of clinics in either study group reporting screening. TB is one of the most common AIDS-indicative diseases diagnosed in the WHO European Region,[4] and HIV clinical protocols for the WHO European Region call for all PLHIV to be screened for TB.[25] Fairly large proportions of EuroSIDA clinics in both East Europe and non-East Europe countries appear to not be implementing this guideline. It is possible that the consequences are more pronounced in the East Europe countries, given the high burden of TB and multidrug-resistant TB in those countries.[26] The low level of integration of TB treatment into HIV care and treatment in the East Europe study region – with only 22% of clinics reporting this to be the case – raises serious concerns in light of what is known about the benefits of an integrated clinical approach to TB/HIV co-infection.[27]

The East Europe clinics lagged behind other clinics in regard to screening for cardiovascular disease, tobacco use, alcohol consumption and drug use, all issues with important implications for PLHIV. Cardiovascular disease is a major cause of non-HIV-related mortality in PLHIV populations,[28,29] and people who smoke tobacco have an elevated risk of cardiovascular disease.[30] Alcohol consumption exacerbates the effects on the liver of HBV, HCV and other liver diseases,[31] as well as potentially having other negative health effects.[32,33] While screening for drug use is considered a good practice in many clinical settings, it warrants special consideration in settings where a major pathway for HIV transmission is injecting drug use. This is the case in the East Europe EuroSIDA clinics, with 38% of the 1,370 East Europe study participants who contributed data to this study reporting injecting drug use as their mode of exposure to HIV. In this context, the reported absence of routine screening for drug use at 42% of East Europe clinics stands out as an issue that may have important implications for patients’ health.

Taken together, our study findings raise important questions regarding whether the availability of a range of services at clinics caring for PLHIV might have an impact on morbidity and mortality. At a time when antiretroviral therapy is widely available in high-income countries and is becoming increasingly available in low- and middle-income countries, these questions are important to take up since they reflect a growing awareness that antiretroviral therapy alone is not sufficient to safeguard the long-term health of PLHIV. Surprisingly, in light of the advanced state of HIV management in some regards, there appears to not be a large evidence base regarding which services a clinic should provide to its HIV-positive patients. The comprehensive HIV care model, with a healthcare team coordinating primary care, HIV care, and other specialist care as well as psychosocial and social services, has long been championed in the United States [34] and has likely influenced the development of many multifaceted HIV clinical initiatives worldwide. However, there is scant evidence regarding the relationship between specific HIV clinic characteristics and patient health outcomes.

A 2006 Cochrane review of studies that assessed various elements of the “setting and organisation of care” for PLHIV found an association between case management and decreased mortality, but concluded that the small evidence base in this field was not sufficient to determine an ideal set of clinic characteristics.[35] Since the publication of the Cochrane review, little new evidence has emerged. A 2009 retrospective cohort study of PLHIV at health facilities for US military veterans found that patients attending clinics that integrated hepatitis, psychiatric, psychological and social services into HIV clinical management were more than three times as likely to achieve viral suppression on ART than patients attending HIV clinics without integrated services.[36]

A key challenge in conceptualising and conducting meaningful research in this domain is the setting-specific nature of how health services are organised, funded, managed and governed. For example, the issue of whether or how to integrate HIV clinical services with other clinical services has been highlighted particularly in the context of efforts to provide HIV care in severely resource-constrained settings, with studies in sub-Saharan Africa and elsewhere examining different service delivery models [37] and undertaking different forms of service integration in order to provide HIV services alongside other services such as tuberculosis management [38] and reproductive health care.[39] This research may be of limited value to health system decision-makers in settings with much higher physician-patient ratios or with strong referral systems linking long-established HIV clinics to other services. Clearly there are major differences in regard to which health service delivery models are predominant in different countries, with some differences largely attributable to resource limitations in poorer countries.

Nonetheless, the identification of key elements of successful patient management in diverse settings presents opportunities to explore ways in which these elements may or may not be uniquely dependent on specific features of the local and national health system and the social, political and economic context. It is entirely possible that some service delivery innovations in sub-Saharan Africa may be relevant to health systems in Western Europe, and vice versa. By calling attention to ways in which two regional groupings of clinics for PLHIV in Europe differ from each other, the EuroSIDA clinic survey findings serve as an invitation for clinics and regions with suboptimal patient outcomes to investigate whether the adoption of practices from other settings may be beneficial. Thus the lack of generalisability of a study of the characteristics of health service delivery in a specific group of clinics may be offset by its potential to highlight issues warranting further setting-specific research including operational research on service delivery modifications in “real world” clinic populations.

In sum, our study makes a unique contribution to the issue of HIV management by exploring whether the characteristics of clinics vary across two groups of European countries that have had marked differences in patient outcomes in a large observational study cohort. The identification of some statistically significant differences in clinic characteristics cannot be interpreted as evidence that one or more of those differences is causing the observed differences in patient outcomes. However, findings suggest that it may be beneficial to conduct further research on the potential health impact of clinic characteristics such as the CD4 threshold for ART initiation; ART resistance testing practices; HCV treatment standards; and the provision of screening for non-HIV-related conditions including alcohol and drug dependency. Policy-makers should consider research on service delivery factors alongside other types of research on biomedical factors, health system factors and the influence of the social, political and economic context in order to optimally configure health care for PLHIV at the national and subnational levels.

**Limitations**

This study has the following limitations. While our survey addressed major aspects of the clinical and non-clinical care of people living with HIV, there may be other aspects of patient care that have implications for patient outcomes. The study utilises survey data that were reported by clinic representatives whose responses to questions might not reflect what actually happens in clinical practice. Clinical decision-making about some of the issues addressed in our study can be expected to vary in accordance with individual physicians’ preferences as well as patient characteristics such as the nature of symptoms and severity of disease. Furthermore, study findings reflect self-reporting, and it is not possible to verify the accuracy of the information reported. Respondents may have made errors or altered their responses to suggest a higher level of compliance with guidelines. The survey was conducted in English, which may have affected how questions were interpreted by some respondents. Findings have limited generalisability because the EuroSIDA clinics constitute only a small proportion of HIV clinics in European countries. Generalisability is also affected by key characteristics of the responding EuroSIDA clinics: half were university clinics, almost half were government-affiliated, and many were located in capital cities. Practices at these clinics are therefore not necessarily representative of HIV management in the European region overall.

**CONCLUSIONS**

Our study findings raise important questions about how specific features of HIV clinics might contribute to the geographical differences in patient outcomes in the EuroSIDA study cohort. Significantly more East Europe clinics deferred ART in asymptomatic patients until the CD4 cell count dropped below 350 cells/mm^3^, and significantly fewer East Europe clinics provided resistance testing before initiating ART or upon ART failure. The East Europe group of clinics also compared unfavourably to the non-East Europe group in regard to HBV vaccination, DAA access, TB/HIV treatment integration and screening for cardiovascular disease, smoking, alcohol use and drug use. There is a need for further research to guide setting-specific decision-making regarding the optimal array of services at HIV clinics in Europe and worldwide.

**DECLARATIONS**

**List of abbreviations**

HIV: Human immunodeficiency virus

ART: antiretroviral therapy

HBV: Hepatitis B

HCV: Hepatitis C

TB: Tuberculosis

DAA: Direct acting antivirals

PHLIV: People living with HIV

WHO: World Health Organization

CHIP: The Centre for Health and Infectious Disease Research

**Ethics and consent to participate**

**The EuroSIDA study collects patient data and the most recent Danish EuroSIDA approval (EuroSIDA coordination office located in Copenhagen, Denmark) was granted on 30 June 2014 with Journal no. H-3-2012-049 by the Research Ethics Committee for the Capital Region of Denmark. Further, ethics approval was obtained in each individual country involved in the study (Additional File 2). A**ll EuroSIDA patients provided informed consent.

**Consent to publish**

Not applicable.

**Competing interests**

The authors have no competing interests to declare.

**Funding**

Primary funding for EuroSIDA is provided by the European Union’s Seventh Framework Programme for research, technological development and demonstration under EuroCoord grant agreement n˚ 260694. Current support also includes unrestricted grants by Bristol-Myers Squibb, Janssen R&D, Merck and Co. Inc., Pfizer Inc. and GlaxoSmithKline LLC. This study was supported by a grant [grant number DNRF126] from the Danish National Research Foundation. The participation of centres from Switzerland was supported by The Swiss National Science Foundation (Grant 108787).

**Authors’ contributions**

JVL and AM conceived the idea of the study and developed the survey with input from KSH. KGL managed the survey implementation. AM carried out the statistical analysis. All authors contributed to the data analysis and interpretation of the results. JVL and KSH drafted the article. All authors have read and approved the final manuscript.

**Availability of data and materials**

**All data supporting the findings are available from the authors upon request.**

**Acknowledgements**

We thank Ole Kirk, CHIP, Rigshospitalet, for his contribution to the survey and his comments on multiple versions of this article. We further thank members of the EuroSIDA multicentre study group (Additional File 3) for their contributions.

**Additional files**

1. EuroSIDA Clinic Survey

2. Ethics committees by country

3. The multi-centre study group, EuroSIDA

**References**

1. Hull MW, Lima VD, Hogg RS, Harrigan PR, Montaner JSG. Epidemiology of treatment failure: a focus on recent trends. Curr Opin HIV AIDS. 2009 Nov;4(6):467–73.

2. Antiretroviral Therapy Cohort Collaboration. Life expectancy of individuals on combination antiretroviral therapy in high-income countries: a collaborative analysis of 14 cohort studies. Lancet. 2008 Jul 26;372(9635):293–9.

3. Samji H, Cescon A, Hogg RS, Modur SP, Althoff KN, Buchacz K, et al. Closing the gap: increases in life expectancy among treated HIV-positive individuals in the United States and Canada. PloS One. 2013;8(12):e81355.

4. European Centre for Disease Prevention and Control/World Health Organization Regional Office for Europe. HIV/AIDS surveillance in Europe 2013 [Internet]. 2014. Available from: http://ecdc.europa.eu/en/publications/Publications/hiv-aids-surveillance-report-Europe-2013.pdf

5. Lozano R, Naghavi M, Foreman K, Lim S, Shibuya K, Aboyans V, et al. Global and regional mortality from 235 causes of death for 20 age groups in 1990 and 2010: a systematic analysis for the Global Burden of Disease Study 2010. The Lancet. 2013;380(9859):2095–128.

6. Global Health Observatory. Number of deaths due to HIV/AIDS: estimates by WHO region [Internet]. [cited 2015 Aug 5]. Available from: http://apps.who.int/gho/data/view.main.22600WHO?lang=en

7. Murray CJL, Ortblad KF, Guinovart C, Lim SS, Wolock TM, Roberts DA, et al. Global, regional, and national incidence and mortality for HIV, tuberculosis, and malaria during 1990-2013: a systematic analysis for the Global Burden of Disease Study 2013. Lancet. 2014 Sep 13;384(9947):1005–70.

8. Islam FM, Wu J, Jansson J, Wilson DP. Relative risk of cardiovascular disease among people living with HIV: a systematic review and meta-analysis. HIV Med. 2012 Sep;13(8):453–68.

9. Rubinstein PG, Aboulafia DM, Zloza A. Malignancies in HIV/AIDS: from epidemiology to therapeutic challenges. AIDS. 2014 Feb 20;28(4):453–65.

10. Masiá M, Padilla S, Álvarez D, López JC, Santos I, Soriano V, et al. Risk, predictors, and mortality associated with non-AIDS events in newly diagnosed HIV-infected patients: role of antiretroviral therapy. AIDS. 2013 Jan 14;27(2):181–9.

11. European AIDS Clinical Society. Guidelines Version 7.1 [Internet]. 2014 [cited 2015 Sep 16]. Available from: http://www.eacsociety.org/files/guidelines-7.1-english.pdf

12. Garcia de la Hera M, Davo MC, Ballester-Añón R, Vioque J. The opinions of injecting drug user (IDUs) HIV patients and health professionals on access to antiretroviral treatment and health services in Valencia, Spain. Eval Health Prof. 2011 Sep;34(3):349–61.

13. Bogart LM, Wagner GJ, Green HD Jr, Mutchler MG, Klein DJ, McDavitt B. Social network characteristics moderate the association between stigmatizing attributions about HIV and non-adherence among Black Americans living with HIV: a longitudinal assessment. Ann Behav Med. 2015 Dec;49(6):865–72.

14. Johnson M, Samarina A, Xi H, et al. Barriers to access to care reported by women living with HIV across 27 countries. AIDS Care. 2015;27(10):1220–30.

15. Bannister WP, Kirk O, Gatell JM, Knysz B, Viard J-P, Mens H, et al. Regional changes over time in initial virologic response rates to combination antiretroviral therapy across Europe. J Acquir Immune Defic Syndr. 2006 Jun;42(2):229–37.

16. Podlekareva DN, Reekie J, Mocroft A, Losso M, Rakhmanova AG, Bakowska E, et al. Benchmarking HIV health care: from individual patient care to health care evaluation. An example from the EuroSIDA study. BMC Infect Dis. 2012;12:229.

17. Reekie J, Kowalska JD, Karpov I, Rockstroh J, Karlsson A, Rakhmanova A, et al. Regional differences in AIDS and non-AIDS related mortality in HIV-positive individuals across Europe and Argentina: the EuroSIDA study. PloS One. 2012;7(7):e41673.

18. Mocroft A, Ledergerber B, Katlama C, Kirk O, Reiss P, d’Arminio Monforte A, et al. Decline in the AIDS and death rates in the EuroSIDA study: an observational study. Lancet. 2003 Jul 5;362(9377):22):22

19. Podlekareva D, Reekie J, Mocroft A, Losso M, Rakhmanova A, Bakowska E, Karpov IA, Lazarus J, Gatell J, Lundgren JD, Kirk O. Benchmarking HIV health care: from individual patient care to health care evaluation. An example from the EuroSIDA study. BMC Infect Dis. 2012 Sep 25;12(1):229.

20. World Health Organization. Consolidated guidelines on the use of antiretroviral drugs for treating and preventing HIV infection: recommendations for a public health approach June 2013 [Internet]. 2013. Available from: http://apps.who.int/iris/bitstream/10665/85321/1/9789241505727_eng.pdf?ua=1

21. Acharya C, Dharel N, Sterling RK. Chronic liver disease in the human immunodeficiency virus patient. Clin Liver Dis. 2015 Feb;19(1):1–22.

22. Graham CS, Swan T. A path to eradication of hepatitis C in low- and middle-income countries. Antiviral Res. 2015 Jul;119:89–96.

23. Ippolito G, Capobianchi MR, Lanini S, Antonelli G. Is hepatitis C virus eradication around the corner only 25 years after its discovery? Int J Antimicrob Agents. 2015 Feb;45(2):111–2.

24. Peters L, et al. HIV/HCV co-infection across Europe. In Barcelona, Spain; 2015.

25. World Health Organization Regional Office for Europe. HIV/AIDS treatment and care: clinical protocols for the WHO European region [Internet]. 2007. Available from: http://www.euro.who.int/__data/assets/pdf_file/0004/78106/E90840.pdf?ua=1

26. European Centre for Disease Prevention and Control. Tuberculosis surveillance and monitoring in Europe 2015 [Internet]. 2015. Available from: http://ecdc.europa.eu/en/publications/Publications/tuberculosis-surveillance-monitoring-Europe-2015.pdf

27. Mansfeld M, Skrahina A, Shepherd L, Schultze A, Panteleev AM, Miller RF, et al. Major differences in organization and availability of health care and medicines for HIV/TB coinfected patients across Europe. HIV Med. 2015 May 11;

28. Palella FJ, Baker RK, Moorman AC, Chmiel JS, Wood KC, Brooks JT, et al. Mortality in the highly active antiretroviral therapy era: changing causes of death and disease in the HIV outpatient study. J Acquir Immune Defic Syndr 1999. 2006 Sep;43(1):27–34.

29. Smith CJ, Ryom L, Weber R, Morlat P, Pradier C, Reiss P, et al. Trends in underlying causes of death in people with HIV from 1999 to 2011 (D:A:D): a multicohort collaboration. Lancet. 2014 Jul 19;384(9939):241–8.

30. Tonstad S, Andrew Johnston J. Cardiovascular risks associated with smoking: a review for clinicians. Eur J Cardiovasc Prev Rehabil Off J Eur Soc Cardiol Work Groups Epidemiol Prev Card Rehabil Exerc Physiol. 2006 Aug;13(4):507–14.

31. Lee M, Kowdley KV. Alcohol’s effect on other chronic liver diseases. Clin Liver Dis. 2012 Nov;16(4):827–37.

32. Grønbaek M. The positive and negative health effects of alcohol- and the public health implications. J Intern Med. 2009 Apr;265(4):407–20.

33. Mukamal K. Alcohol consumption: risks and benefits. Curr Atheroscler Rep. 2008;10(6):536–43.

34. Gallant JE, Adimora AA, Carmichael JK, Horberg M, Kitahata M, Quinlivan EB, et al. Essential components of effective HIV care: a policy paper of the HIV Medicine Association of the Infectious Diseases Society of America and the Ryan White Medical Providers Coalition. Clin Infect Dis Off Publ Infect Dis Soc Am. 2011 Dec;53(11):1043–50.

35. Handford CD, Tynan AM, Rackal JM, Glazier RH. Setting and organization of care for persons living with HIV/AIDS. Cochrane Database Syst Rev. 2006;(3):CD004348.

36. Hoang T, Goetz MB, Yano EM, Rossman B, Anaya HD, Knapp H, et al. The impact of integrated HIV care on patient health outcomes. Med Care. 2009 May;47(5):560–7.

37. Uebel KE, Joubert G, Wouters E, Mollentze WF, van Rensburg DHCJ. Integrating HIV care into primary care services: quantifying progress of an intervention in South Africa. PloS One. 2013;8(1):e54266.

38. Legido-Quigley H, Montgomery CM, Khan P, Atun R, Fakoya A, Getahun H, et al. Integrating tuberculosis and HIV services in low- and middle-income countries: a systematic review. Trop Med Int Health. 2013 Feb;18(2):199–211.

39. Kennedy CE, Spaulding AB, Brickley DB, Almers L, Mirjahangir J, Packel L, et al. Linking sexual and reproductive health and HIV interventions: a systematic review. J Int AIDS Soc. 2010;13:26.

**Figure 1. Regional differences in ART treatment and management**

(1) data available for N=79, 12 from East Europe and 67 from non-East Europe

**Figure 2. Regional differences in management of hepatitis B and hepatitis C**

*including yes and sometimes

(1) data available for N=78, 12 from East Europe and 66 from non-East Europe

**Figure 3. Regional differences in management of tuberculosis**

(1) relating to one question; data available for N=78, 11 from East Europe and 67 from non-East Europe

**Figure 4. Regional differences in routine screening for other health issues**

(1) data available for N=76, 12 from East Europe and 64 from non-East Europe

(2) data available for N=78, 12 from East Europe and 66 from non-East Europe

(3) data available for N=80, 12 from East Europe and 68 from non-East Europe

**Figure 5. Regional differences in non-clinical support services**

(1) data available for N=80, 12 from East Europe and 68 from non-East Europe

(2) data available for N=79, 11 from East Europe and 68 from non-East Europe

(3) data available for N=76, 11 from East Europe and 65 from non-East Europe

(4) data available for N=80, 12 from East Europe and 68 from non-East Europe

(A) Of 27 clinics answering yes, the median % LTFU was 15 (IQR 12–20) in East Europe and 10 (IQR 8–11) in non-East Europe; p=0.021
