# Supplementary material for: Disparities in HIV clinic care across Europe: findings from the EuroSIDA clinic survey
Source: BMC Infect Dis. 2016 Jul 20;16:335. doi: 10.1186/s12879-016-1685-x (PMC4955207; doi:10.1186/s12879-016-1685-x)
Supplement: Additional file 2: — The multi-centre EuroSIDA study group. (DOCX 29 kb) [file 12879_2016_1685_MOESM2_ESM.docx]

The multi-centre study group, EuroSIDA (national coordinators in parenthesis).

Argentina: (M Losso), M Kundro, Hospital JM Ramos Mejia, Buenos Aires.

Austria: (N Vetter), Pulmologisches Zentrum der Stadt Wien, Vienna; R Zangerle, Medical University Innsbruck, Innsbruck.

Belarus: (I Karpov), A Vassilenko, Belarus State Medical University, Minsk, VM Mitsura, Gomel State Medical University, Gomel; D Paduto, Regional AIDS Centre, Svetlogorsk.

Belgium: (N Clumeck), S De Wit, M Delforge, Saint-Pierre Hospital, Brussels; E Florence, Institute of Tropical Medicine, Antwerp; L Vandekerckhove, University Ziekenhuis Gent, Gent.

Bosnia-Herzegovina: (V Hadziosmanovic), Klinicki Centar Univerziteta Sarajevo, Sarajevo.

Bulgaria: (K Kostov), Infectious Diseases Hospital, Sofia.

Croatia: (J Begovac), University Hospital of Infectious Diseases, Zagreb.

Czech Republic: (L Machala), D Jilich, Faculty Hospital Bulovka, Prague; D Sedlacek, Charles University Hospital, Plzen.

Denmark: G Kronborg,T Benfield, Hvidovre Hospital, Copenhagen; J Gerstoft, T Katzenstein, Rigshospitalet, Copenhagen; NF Møller, C Pedersen, Odense University Hospital, Odense; L Ostergaard, Skejby Hospital, Aarhus, U B Dragsted, Roskilde Hospital, Roskilde; L N Nielsen, Hillerod Hospital, Hillerod.

Estonia: (K Zilmer), West-Tallinn Central Hospital, Tallinn; Jelena Smidt, Nakkusosakond Siseklinik, Kohtla-Järve.

Finland: (M Ristola), I Aho, Helsinki University Central Hospital, Helsinki.

France: (J-P Viard), Hôtel-Dieu, Paris; P-M Girard, Hospital Saint-Antoine, Paris; L Cotte, Hôpital de la Croix Rousse, Lyon; C Pradier, E Fontas, Hôpital de l'Archet, Nice; F Dabis, D Neau, Unité INSERM, Bordeaux, C Duvivier, Hôpital Necker-Enfants Malades, Paris.

Germany: (J Rockstroh), Universitäts Klinik Bonn; R Schmidt, Medizinische Hochschule Hannover; O Degen, University Medical Center Hamburg-Eppendorf, Infectious Diseases Unit, Hamburg; HJ Stellbrink, IPM Study Center, Hamburg; C Stefan, JW Goethe University Hospital, Frankfurt; J Bogner, Medizinische Poliklinik, Munich; G. Fätkenheuer, Universität Köln, Cologne.

Georgia: (N Chkhartishvili) Infectious Diseases, AIDS & Clinical Immunology Research Center, Tbilisi

Greece: (J Kosmidis), P Gargalianos, G Xylomenos, P Lourida, Athens General Hospital; H Sambatakou, Ippokration General Hospital, Athens.

Hungary: (J Szlávik), Szent Lásló Hospital, Budapest.

Iceland: (M Gottfredsson), Landspitali University Hospital, Reykjavik.

Ireland: (F Mulcahy), St. James's Hospital, Dublin.

Israel: (I Yust), D Turner, M Burke, Ichilov Hospital, Tel Aviv; E Shahar, G Hassoun, Rambam Medical Center, Haifa; H Elinav, M Haouzi, Hadassah University Hospital, Jerusalem; D Elbirt, ZM Sthoeger, AIDS Center (Neve Or), Jerusalem.

Italy: (A D’Arminio Monforte), Istituto Di Clinica Malattie Infettive e Tropicale, Milan; R Esposito, I Mazeu, C Mussini, Università Modena, Modena; F Mazzotta, A Gabbuti, Ospedale S Maria Annunziata, Firenze; V Vullo, M Lichtner, University di Roma la Sapienza, Rome; M Zaccarelli, A Antinori, R Acinapura, M Plazzi, Istituto Nazionale Malattie Infettive Lazzaro Spallanzani, Rome; A Lazzarin, A Castagna, N Gianotti, Ospedale San Raffaele, Milan; M Galli, A Ridolfo, Osp. L. Sacco, Milan.

Latvia: (B Rozentale), Infectology Centre of Latvia, Riga.

Lithuania: (V Uzdaviniene) Vilnius University Hospital Santariskiu Klinikos, Vilnius; R Matulionyte, Center of Infectious Diseases, Vilnius University Hospital Santariskiu Klinikos, Vilnius.

Luxembourg: (T Staub), R Hemmer, Centre Hospitalier, Luxembourg.

Netherlands: (P Reiss), Academisch Medisch Centrum bij de Universiteit van Amsterdam, Amsterdam.

Norway: (V Ormaasen), A Maeland, J Bruun, Ullevål Hospital, Oslo.

Poland: (B Knysz), J Gasiorowski, M Inglot, Medical University, Wroclaw; A Horban, E Bakowska, Centrum Diagnostyki i Terapii AIDS, Warsaw; R Flisiak, A Grzeszczuk, Medical University, Bialystok; M Parczewski, M Pynka, K Maciejewska, Medical Univesity, Szczecin; M Beniowski, E Mularska, Osrodek Diagnostyki i Terapii AIDS, Chorzow; T Smiatacz, M Gensing, Medical University, Gdansk; E Jablonowska, E Malolepsza, K Wojcik, Wojewodzki Szpital Specjalistyczny, Lodz; I Mozer-Lisewska, Poznan University of Medical Sciences, Poznan.

Portugal: (M Doroana), L Caldeira, Hospital Santa Maria, Lisbon; K Mansinho, Hospital de Egas Moniz, Lisbon; F Maltez, Hospital Curry Cabral, Lisbon.

Romania: (R Radoi), C Oprea, Spitalul de Boli Infectioase si Tropicale: Dr. Victor Babes, Bucarest.

Russia: (A Rakhmanova) (deceased), Medical Academy Botkin Hospital, St Petersburg; A Rakhmanova, St Petersburg AIDS Centre, St Peterburg; T Trofimora, Novgorod Centre for AIDS, Novgorod, I Khromova, Centre for HIV/AIDS & and Infectious Diseases, Kaliningrad; E Kuzovatova, Nizhny Novgorod Scientific and Research Institute, Nizhny Novogrod.

Serbia: (D Jevtovic), The Institute for Infectious and Tropical Diseases, Belgrade.

Slovakia: A Shunnar, D Staneková, Dérer Hospital, Bratislava.

Slovenia: (J Tomazic), University Clinical Centre Ljubljana, Ljubljana.

Spain: (JM Gatell), JM Miró, Hospital Clinic Universitari de Barcelona, Barcelona; S Moreno, J. M. Rodriguez, Hospital Ramon y Cajal, Madrid; B Clotet, A Jou, R Paredes, C Tural, J Puig, I Bravo, Hospital Germans Trias i Pujol, Badalona; P Domingo, M Gutierrez, G Mateo, MA Sambeat, Hospital Sant Pau, Barcelona; JM Laporte, Hospital Universitario de Alava, Vitoria-Gasteiz.

Sweden: (K Falconer), A Thalme, A Sonnerborg, Karolinska University Hospital, Stockholm; A Blaxhult, Venhälsan-Sodersjukhuset, Stockholm; L Flamholc, Malmö University Hospital, Malmö.

Switzerland: (B Ledergerber), R Weber, University Hospital Zurich; M Cavassini, University Hospital Lausanne; A Calmy, University Hospital Geneva; H Furrer, University Hospital Bern; M Battegay, University Hospital Basel; P Schmid, Cantonal Hospital St. Gallen.

Ukraine: (E Kravchenko), Kiev Centre for AIDS, Kiev; V Frolov, G Kutsyna, I Baskakov, Luhansk State Medical University, Luhansk; A Kuznetsova, Kharkov State Medical University, Kharkov; G Kyselyova, Crimean Republican AIDS centre, Simferopol; M Sluzhynska, Lviv Regional HIV/AIDS Prevention and Control CTR, Lviv.

United Kingdom: (B Gazzard), St. Stephen's Clinic, Chelsea and Westminster Hospital, London; AM Johnson, E Simons, S Edwards, Mortimer Market Centre, London; A Phillips, MA Johnson, A Mocroft, Royal Free and University College Medical School, London (Royal Free Campus); C Orkin, Royal London Hospital, London; J Weber, G Scullard, Imperial College School of Medicine at St. Mary's, London; A Clarke, Royal Sussex County Hospital, Brighton; C Leen, Western General Hospital, Edinburgh.

The following centers have previously contributed data to EuroSIDA:

Hôpital de la Pitié-Salpétière, Paris, France

Bernhard Nocht Institut für Tropenmedizin, Hamburg, Germany

1st I.K.A Hospital of Athens, Athens, Greece

Ospedale Riuniti, Divisione Malattie Infettive, Bergamo, Italy

Ospedale di Bolzano, Divisione Malattie Infettive, Bolzano, Italy

Ospedale Cotugno, III Divisione Malattie Infettive, Napoli, Italy

Hospital Carlos III, Departamento de Enfermedades Infecciosas, Madrid, Spain

Odessa Region AIDS Center, Odessa, Ukraine

EuroSIDA Steering Committee

Steering Committee: : J Gatell, B Gazzard, A Horban, I Karpov, B Ledergerber, M Losso, A d’Arminio Monforte, C Pedersen, A Rakhmanova, M Ristola, A Phillips, P Reiss, J Lundgren, J Rockstroh, S De Wit

Chair: J Rockstroh

Vice-chair: S De Wit

Study Co-leads: A Mocroft, O Kirk

EuroSIDA Representatives to EuroCoord: O Kirk, A Mocroft, J Grarup, P Reiss, A Cozzi-Lepri, R Thiebaut, J Rockstroh, D Burger, R Paredes, L Peters

EuroSIDA staff

Coordinating Centre Staff: O Kirk, L Peters, C Matthews, AH Fischer, A Bojesen, D Raben, D Kristensen, K Grønborg Laut, JF Larsen, D Podlekareva

Statistical Staff: A Mocroft, A Phillips, A Cozzi-Lepri, L Shepherd, A Schultze

Funding

Primary funding for EuroSIDA is provided by the European Union’s Seventh Framework Programme for research, technological development and demonstration under EuroCoord grant agreement n˚ 260694. Current support also includes unrestricted grants by Bristol-Myers Squibb, Janssen R&D, Merck and Co. Inc., Pfizer Inc., GlaxoSmithKline LLC. The participation of centres from Switzerland was supported by The Swiss National Science Foundation (Grant 108787).
