# Supplementary material for: Disparities in HIV clinic care across Europe: findings from the EuroSIDA clinic survey
Source: BMC Infect Dis. 2016 Jul 20;16:335. doi: 10.1186/s12879-016-1685-x (PMC4955207; doi:10.1186/s12879-016-1685-x)
Supplement: Additional file 3: — Ethics committees by country. (PDF 110 kb) [file 12879_2016_1685_MOESM3_ESM.pdf]

EuroSIDA ethics committees

Country	Ethics committee
Belarus	Health Committee of Minsk City Executive
Estonia	Tallinn Medical Research Ethics Committee
Lithuania	Vilniaus Regioninis Biomedicininių Tyrimų Etikos Komitetas
Russia	Independent Ethics Committee at GBUZ SO "Samara City Hospital #4"
Ukraine	License of the Kharkiv Regional Centre for Disease Prevention and Control of AIDS Medical Practice
Denmark	De Videnskabsetiske Komiteer for Region Hovedstaden
Finland	Helsingin Ja Uudenmaan Sairaanhoitopiiri
Iceland	Visindasiđanefnd
Ireland	SJH/AMNCH Research Ethics Committee
Netherlands	Covered by the ATHENA cohort
Norway	Regionale Komiteer For Medisinsk Og Helsefaglig Forskningsetikk
Sweden	Regionala etikprövningsnämden i Stockholm
United Kingdom	Research and Development Office
Greece	Georgios Gennimatas General State Hospital, Scientific Committee'
Israel	The Committee on Research Involving Human Subjects of the Hebrew University-Hadassah Medical School
Italy	Comitato Etico Provinciale
Portugal	Hospital De Santamaria
Spain	Secretaria Del Comité Ètico De Investigación Clinica (CEIC)
Austria	Ethikkommission Der Stadt Wien
Belgium	Comite Local D'ethique Hospitalier
France	Does not require ethics committee approval
Germany	Ethikkommission Bei Der Lmu München
Luxembourg	Comité National D'éthique De Recherche
Switzerland	Kantonale Ethikkommission (Kek)
Croatia	University Hospital For Infectious Diseases Ethics Committee
Czech Republic	Fakultni Nemocnice Na Bulovce Eticka Komise
Hungary	Intézeti Kutatásetikai Bizottság
Poland	Komisja Bioetyki Uniwersytetu Medycznego W Lodzi
Romania	Comisia Locala De Etica A Spitalului De Boli Infectioase Si Tropicala
Serbia	Klinicki Centar Srbije Eticki Odbor
Slovenia	The National Medical Ethics Committee
